# The Anthelmintic Drug Niclosamide and Its Analogues Activate the Parkinson's Disease Associated Protein Kinase PINK1

**DOI:** 10.1002/cbic.201700500

**Published:** 2018-01-24

**Authors:** Erica Barini, Ageo Miccoli, Federico Tinarelli, Katie Mulholland, Hachemi Kadri, Farhat Khanim, Laste Stojanovski, Kevin D. Read, Kerry Burness, Julian J. Blow, Youcef Mehellou, Miratul M. K. Muqit

**Affiliations:** ^1^ MRC Protein Phosphorylation and Ubiquitylation Unit University of Dundee Dow Street Dundee DD1 5EH UK; ^2^ School of Pharmacy and Pharmaceutical Sciences Cardiff University King Edward VII Avenue Cardiff CF10 3NB UK; ^3^ Centre for Gene Regulation and Expression University of Dundee Dow Street Dundee DD1 5EH UK; ^4^ School of Biosciences University of Birmingham Edgbaston Birmingham B15 2TT UK; ^5^ Drug Discovery Unit University of Dundee Dow Street Dundee DD1 5EH UK; ^6^ MRC PPU Reagents and Services University of Dundee Dow Street Dundee DD1 5EH UK; ^7^ School of Medicine University of Dundee Dow Street Dundee DD1 9SY UK

**Keywords:** drug discovery, membranes, mitochondria, neurodegenerative diseases, proteins

## Abstract

Mutations in PINK1, which impair its catalytic kinase activity, are causal for autosomal recessive early‐onset Parkinson's disease (PD). Various studies have indicated that the activation of PINK1 could be a useful strategy in treating neurodegenerative diseases, such as PD. Herein, it is shown that the anthelmintic drug niclosamide and its analogues are capable of activating PINK1 in cells through the reversible impairment of the mitochondrial membrane potential. With these compounds, for the first time, it is demonstrated that the PINK1 pathway is active and detectable in primary neurons. These findings suggest that niclosamide and its analogues are robust compounds for the study of the PINK1 pathway and may hold promise as a therapeutic strategy in PD and related disorders.

Loss‐of‐function mutations in the genes encoding the phosphatase and tensin homologue deleted on chromosome 10 (PTEN)‐induced kinase 1 (PINK1) and the E3 ubiquitin ligase Parkin lead to autosomal recessive early‐onset Parkinson's disease (PD).^1^ PINK1 is a serine/threonine protein kinase that possesses an N‐terminal mitochondrial targeting sequence, a transmembrane domain, and three insertional loops within its catalytic kinase domain.^2^ A large body of cell biological and biochemical analyses has linked PINK1 to the regulation of mitochondrial homoeostasis.^3^ Indeed, it is now understood that, upon mitochondrial membrane depolarisation, PINK1 becomes activated and, consequently, phosphorylates Parkin and ubiquitin at a conserved residue (Ser65). This stimulates Parkin recruitment to the mitochondria, whereupon it becomes maximally active and ubiquitylates multiple substrates on the outer mitochondrial membrane to trigger degradation of damaged mitochondria through autophagy (mitophagy).^4^

The majority of PD‐related PINK1 mutations abrogate its kinase activity^5^ and prevent the initiation of mitophagy in cells upon mitochondrial damage, leading to the accumulation of reactive oxygen species and premature neuronal loss.^6^ This underlines the kinase activity of PINK1 as being critical to the prevention of neurodegeneration. Such a hypothesis has been verified in Drosophila models of PINK1, in which kinase‐inactive versions of PINK1 failed to rescue neurodegeneration relative to that of the wild‐type (WT) gene.^7^ This important finding highlighted the activation of PINK1 as a promising strategy for inducing and maintaining neuroprotective effects.

To date, a series of agents have been reported to efficiently activate PINK1 in various immortalised human cell lines. These could be divided into two groups: compounds that act directly as PINK1 ATP neosubstrates^8^ and indirect PINK1 activators that cause the loss of the mitochondrial membrane potential (Δ*ψm*).^9^ Undoubtedly, the latter class of compounds, which include the proton ionophore, carbonyl cyanide *m*‐chlorophenyl hydrazone (CCCP), the potassium uniporter valinomycin, or a combination of antimycin A and oligomycin A (A/O) have attracted more interest in the study of PINK1 signalling. Despite the promise of these agents in activating PINK1, their cellular toxicity has limited their translation to activating PINK1 in vivo. Hence, the elaboration of novel and safe (direct or indirect) activators of PINK1 is of great biological and therapeutic interest.

Because indirect PINK1 activation can be triggered by the uncoupling of the mitochondria,^9^ we focused our search for small‐molecule PINK1 activators on niclosamide (Figure 1 A); an anthelminthic drug previously reported for its potential in treating myeloma through the uncoupling of oxidative phosphorylation in the mitochondria.^10^ Given that niclosamide has been used for a long time as a safe anthelminthic drug^11^ and studied in vivo with no apparent severe side effects,^12^ we were encouraged to explore the activation of PINK1 by this clinical agent. Accordingly, untagged Parkin was expressed in both WT and PINK1 knockout HeLa cells generated by TALEN technology.^13^ The cells were treated with different concentrations of niclosamide (0.2–20 μm) for 40 min, DMSO or 10 μm/1 μm A/O for 3 h. The cell lysates were immunoblotted with an anti‐phospho‐Parkin Ser65 antibody to monitor PINK1 activity.4c Niclosamide has been shown to uncouple the mitochondria to prevent the creation of adenosine triphosphate (ATP).12b To monitor the ability of niclosamide and A/O to induce mitochondrial uncoupling, we probed the cleavage of the mitochondrial protein, optic atrophy protein 1 (OPA1), that is catalysed by the zinc metalloprotease, OMA1, upon mitochondrial membrane depolarisation in cells.^14^ We observed mild activation of PINK1, as determined by Parkin Ser65 phosphorylation at 0.2 μm, and more striking activation at 2 μm or higher concentrations of niclosamide comparable to that induced by A/O treatment at 3 h (Figures 1 B). This was associated with ubiquitylation of the mitochondrial Fe/S domain‐containing protein, CISD1, that is a readout of Parkin ubiquitin E3 ligase activity (Figure 1 B).^15^

**Figure 1:**
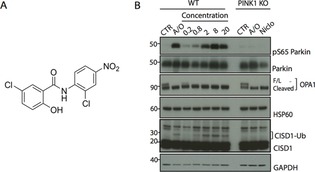
Niclosamide activates PINK1 in HeLa cells. A) Chemical structure of niclosamide. B) Niclosamide dose–response analysis. WT and PINK1 knockout (PINK1 KO) HeLa cells transfected with Parkin were stimulated with either a combination of A/O for 3 h or with different concentrations (0.2, 0.8, 2, 8, 20 μm) of niclosamide (Niclo) for 40 min. Parkin Ser65 phosphorylation (pS65Parkin), Parkin, Full length OPA1 (F/L), Cleaved OPA1, ubiquitylated CISD1 (CISD1‐Ub) and CISD1 were detected by immunoblotting. GAPDH was used as a loading control.

Importantly, the ability of niclosamide and A/O to induce Parkin Ser65 phosphorylation and CISD1 ubiquitylation was abolished in PINK1 knockout cells (Figure 1 B). However, their ability to induce uncoupling was not affected, as determined by cleavage of OPA1 (Figure 1 B). Under similar transfection and cell conditions, we next undertook a time‐course analysis of Parkin Ser65 phosphorylation and CISD1 ubiquitylation in the presence of 20 μm niclosamide. We observed robust niclosamide‐induced Parkin Ser65 phosphorylation after 20 min of treatment (Figure S1 in the Supporting Information) associated with ubiquitylation of CISD1 (Figure S1 A). In vitro kinase assays of PINK1 in the presence or absence of niclosamide showed no evidence of direct activation of PINK1 by the compound (data not shown).

Facile chemical modification of the salicynalide scaffold of niclosamide enabled synthesis of three brominated analogues known to exert pharmacological efficacy, AM85 (Dibromsalan), AM86 (Tribromsalan) and AM87 (Metabromsalan; Figure 2 A; also see the Supporting Information).^16^ To compare the effects of these niclosamide analogues on PINK1 activation, HeLa cells were treated with 20 μm niclosamide or AM85–AM87 for 40 min and this revealed AM85 to be the most potent analogue, as determined by Parkin Ser65 phosphorylation and CISD1 ubiquitylation (Figure 2 B). Interestingly, less potent PINK1 activators AM86 and AM87 induced similar cleavage of OPA1 to A/O and niclosamide, which indicates broadly similar effects on Δ*ψm* (Figure 2 B). We next undertook a dose–response analysis of AM85 on PINK1 activation and observed robust Parkin Ser65 phosphorylation and CISD1 ubiquitylation at 2, 8, and 20 μm, but not at lower concentrations (Figure 2 C). CISD1 ubiquitylation was also confirmed by pull down of ubiquitylated substrates with HALO‐UBQLN1 resin, as previously described (Figure S2 A).4c Furthermore, time‐course analysis of AM85 demonstrated Parkin Ser65 phosphorylation and CISD1 ubiquitylation at 40 min, but not at earlier time points that were previously observed for niclosamide (Figure S2 B and C).

**Figure 2:**
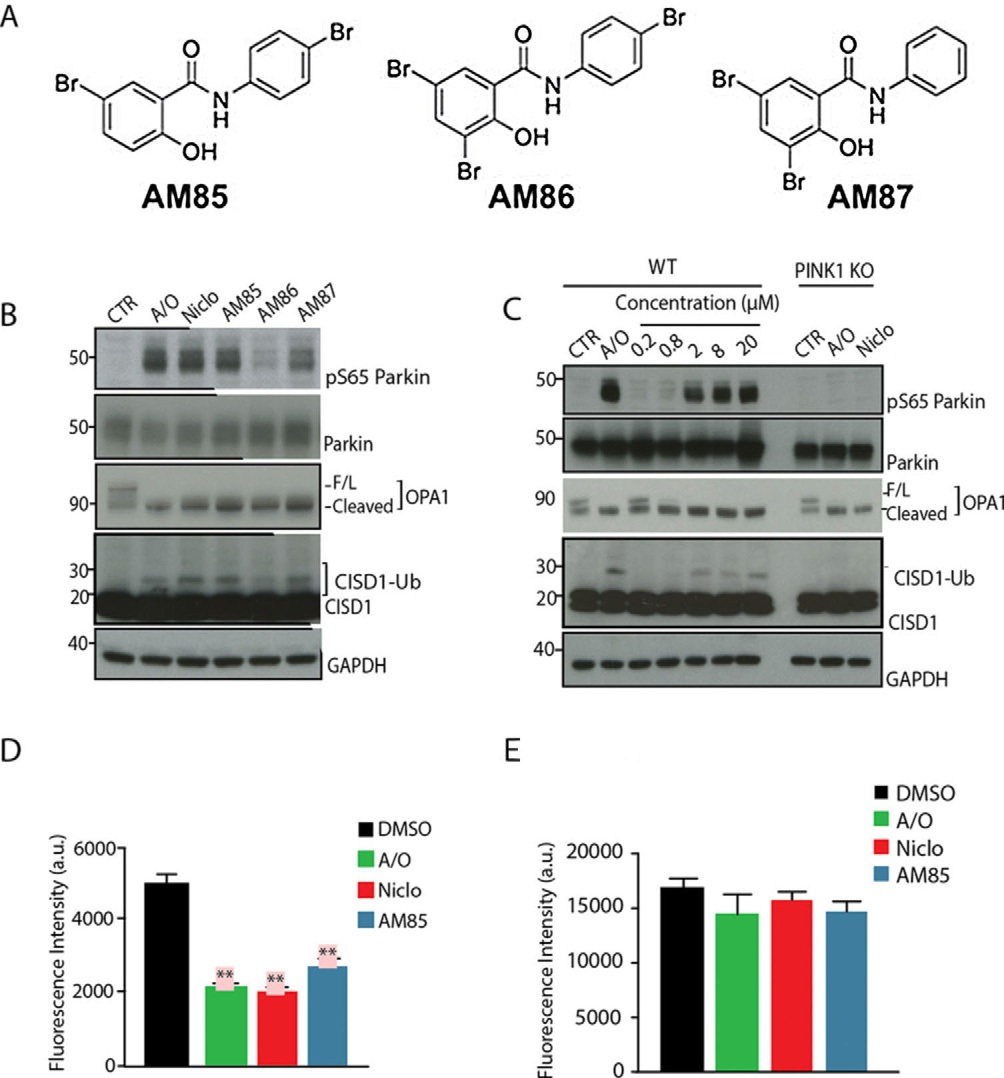
Niclosamide analogue AM85 activates PINK1 in HeLa cells and uncouples mitochondria. A) Chemical structures of niclosamide analogues AM85, AM86, and AM87. B) WT HeLa cells transfected with Parkin were stimulated with either a combination of A/O for 3 h or 10 μm niclosamide (Niclo), AM85, AM86, AM87, for 40 min. C) WT and PINK1 knockout (PINK1 KO) HeLa cells transfected with Parkin were stimulated with A/O for 3 h or with different concentrations (0.2, 0.8, 2, 8, 20 μm) of AM85 for 40 min. Parkin Ser65 phosphorylation (pS65Parkin), Parkin, full‐length OPA1 (F/L), cleaved OPA1, ubiquitylated CISD1 (CISD1‐Ub), and CISD1 were detected by immunoblotting. Glyceraldehyde 3‐phosphate dehydrogenase (GAPDH) was used as a loading control. D) Histograms of CMXRos relative fluorescence intensity [arbitrary units] for HeLa cells treated on site for 3 h with A/O (green) or for 1 h with niclosamide (Niclo, red) and AM85 (blue). Data are normalized to the vehicle DMSO set at 1 (black). E) Quantification of CMXRos relative fluorescence intensity [a.u.] for HeLa cells subjected to drug wash out, after treatment with A/O (green), niclosamide (Niclo, red), AM85 (blue). Data are normalized to the vehicle DMSO set at 1 (black). Bars represent the average ratio±SEM of three independent experiments. ** *p*<0.01, one‐way analysis of variance (ANOVA) followed by Bonferroni post‐test correction.

To quantify the degree of mitochondrial uncoupling between AM85 and niclosamide, we performed fluorescence‐activated cell sorting (FACS) analysis on HeLa cells treated with 20 μm niclosamide and AM85 for 40 min. Cells were incubated with CMX ROS, which is a cell‐permeable probe that accumulates in active mitochondria and emits at *λ*=599 nm.^17^ Interestingly, both niclosamide and AM85 promoted significant mitochondrial membrane depolarisation, comparable to that of 3 h A/O‐treated cells (Figures 2 D and S2 D), which was in agreement with OPA1 cleavage shown above (Figures 1 B and 2 B and C). Critically, the mitochondrial depolarisation effect induced by both niclosamide and AM85 was reversible because no reduction in ROS was detected after compound wash out (Figure 2 E). FACS analysis also demonstrated no significant toxicity under the compound conditions used for niclosamide and AM85, relative to those of DMSO (Figure S3).

We next determined the ability of niclosamide and AM85 to activate PINK1 in cells of pathophysiological relevance to PD. To date, no studies have assessed PINK1 activity in primary neurons under conditions at which PINK1 and Parkin are expressed at endogenous levels. Marked neuronal loss and Lewy body accumulation occurs in the frontal cortex, particularly the anterior cingulate gyrus in advancing PD.^18^ Therefore, we studied primary cortical neurons derived from E16.5 embryos. We initially evaluated Parkin expression in cortical neurons at various time points from 3 to 21 days in vitro (DIV; Figure 3 A). We strikingly observed an increase in Parkin expression during cortical neuronal growth in vitro that paralleled the expression of the pre‐ and post‐synaptic proteins, synaptophysin and PSD95, respectively (Figure 3 A).

**Figure 3:**
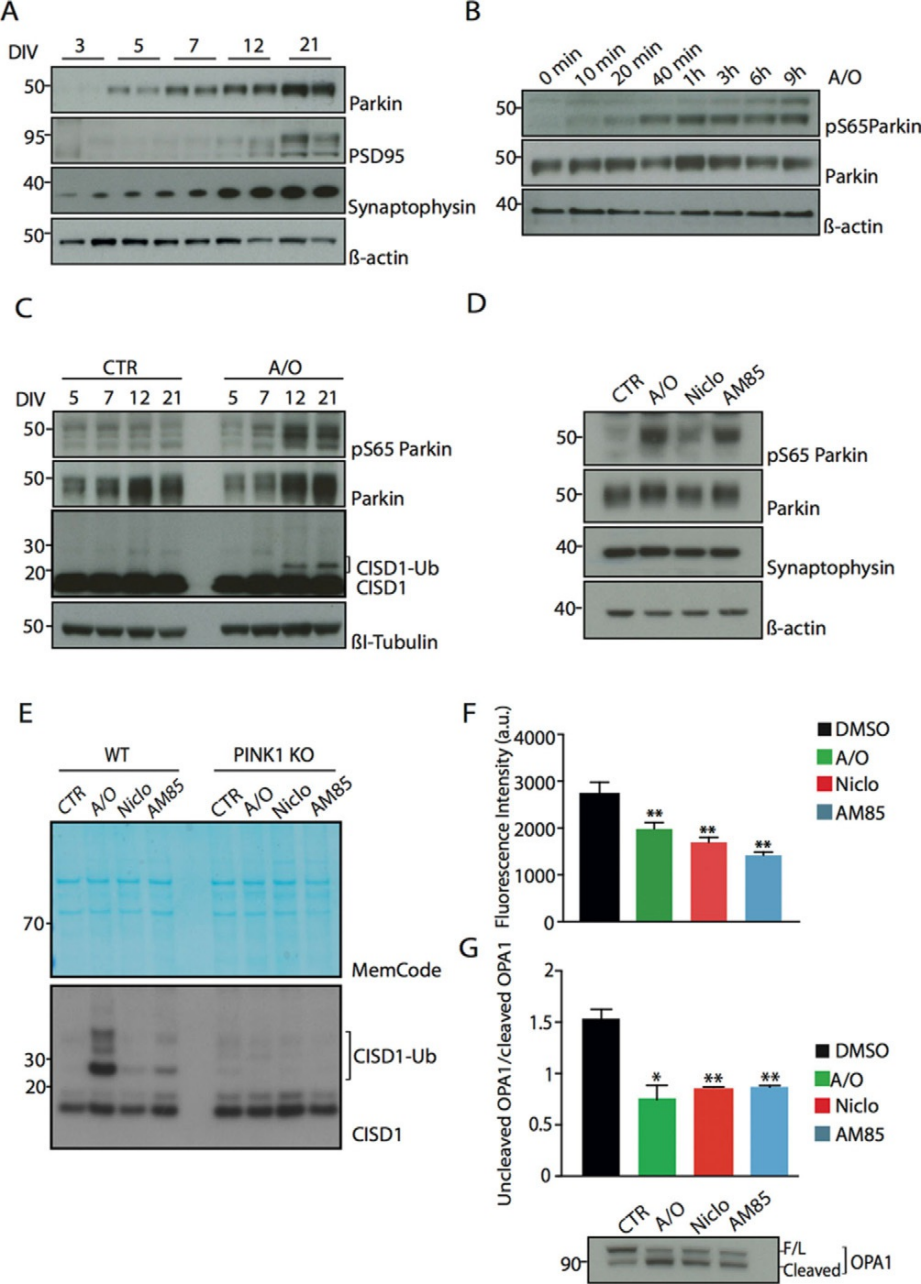
Niclosamide and analogue AM85 activate PINK1 and uncouple mitochondria in primary cortical neurons. A) Detection of Parkin expression at different DIV after plating. PSD95 and synaptophysin were used as markers of increasing neuronal complexity. β‐Actin is used as a loading control. B) Time‐course analysis of Parkin Ser65 phosphorylation (pS65Parkin) and Parkin upon A/O stimulation (10 min, 20 min, 40 min, 1 h, 3 h, 6 h, and 9 h) in 12 DIV neurons. C) E16.5‐derived C57Bl/6J primary cortical neurons after 5, 7, 12, and 21 DIV were stimulated with A/O for 3 h. Parkin Ser65 phosphorylation (pS65Parkin) and CISD1 ubiquitylation (CISD1‐Ub) were detected. βI‐Tubulin was used as a loading control. D) 21 DIV C57Bl/6J primary cortical neurons were stimulated with either a combination of 10 μm A/O for 3 h or 30 μm niclosamide (Niclo) and AM85 for 1 h. Parkin Ser65 phosphorylation (pS65Parkin) and Parkin were detected upon A/O stimulation. E) Pull‐down of ubiquitylated CISD1 (CISD1‐Ub) after niclosamide and AM85 treatment of PINK1 WT and KO 21DIV neurons. MemCode was used as a loading control. F) Graph of CMX ROS fluorescence intensity [a.u.] in 21DIV neurons after treatment with A/O (green), niclosamide (Niclo, red), AM85 (blue), normalised to the vehicle, and DMSO (black). In all graphs, bars represent the average ratio±SEM of two independent experiments. * *p*<0.05, ** *p*<0.01, one‐way ANOVA followed by Bonferroni post‐test correction. G) Niclosamide and AM85 treatment induce OPA1 cleavage. Quantification of cleaved OPA1 in AM85, niclosamide, and A/O treated 21 DIV neurons. Bars represent the average ratio±neurons. Bars represent the average ratio±SEM of two independent experiments and values are ratios between uncleaved and cleaved OPA1. ** *p*<0.01, one‐way ANOVA followed by Bonferroni post‐test correction. At the bottom, exemplary immunoblot of full length (F/L) and cleaved forms of OPA1.

We next undertook a time‐course analysis of 12 DIV neurons treated with A/O, and observed Parkin Ser65 phosphorylation occurring at 10 min of stimulation and becoming maximal by 1 h of stimulation and sustained for 9 h (Figure 3 B). Furthermore, we observed significant CISD1 ubiquitylation in 12 and 21 DIV neurons stimulated with A/O for 3 h, but not in 5 or 7 DIV neurons (Figure 3 C). We next tested the ability of niclosamide and AM85 to activate PINK1 in 21 DIV neurons. Neurons were treated with 30 μm niclosamide or AM85 for 1 h, and this led to increased Parkin Ser65 phosphorylation (Figure 3 D). Interestingly, AM85 exerted a stronger effect on Parkin Ser65 phosphorylation than that of niclosamide; this was also confirmed by CISD1 ubiquitylation in neurons under similar conditions (Figure 3 E). This difference was not explained by differences in uncoupling because both drugs had similar effects on mitochondrial depolarisation, as assessed directly by FACS or indirectly by cleavage of OPA1 (Figure 3 F and G).

In summary, we reported the discovery that the anthelminthic drug, niclosamide, and its analogue, AM85, can activate PINK1 in cells. Notably, we detected, for the first time, PINK1–Parkin pathway activation in neurons and demonstrated that it could be triggered by small molecules. Additionally, we showed that the induction of mitochondrial depolarisation was capable of activating endogenous PINK1 protein in neurons, leading to Parkin Ser65 phosphorylation and ubiquitylation of its mitochondrial substrate CISD1. The mechanism of action of niclosamide and AM85 appears to be indirect and mediated by their mitochondrial uncoupling properties, although this is not sufficient to explain the greater potency of AM85 in neurons. Niclosamide has been used safely in humans for over half a century to treat helminth infections and is currently being tested in multiple clinical trials in a variety of human cancers, as well as rheumatoid arthritis.^19^ Our data suggests that niclosamide and/or its analogues could have therapeutic benefit in slowing down PD progression through the activation of PINK1. Further in vivo studies in appropriate PD models are warranted to test this hypothesis.

## Experimental Section

**Immunoblotting and immunoprecipitation**: Tissues, primary cortical neurons or HeLa cells were sonicated in lysis buffer containing Tris**⋅**HCl (50 mm, pH 7.5), EDTA (1 mm), ethylene glycol bis(β‐aminoethyl ether)‐*N*,*N*,*N*′,*N*′‐tetraacetic acid (EGTA; 1 mm), Triton (1 %, w/v), sodium orthovanadate (1 mm), sodium glycerophosphate (10 mm), sodium fluoride (50 mm), sodium pyrophosphate (10 mm), sucrose (0.25 m), benzamidine (1 mm), phenylmethylsulfonyl fluoride (PMSF; 0.1 mm) and protease inhibitor cocktail (Roche). Following sonication, lysates were incubated for 30 min on ice. Samples were spun at 20 800 *g* in an Eppendorf 5417R centrifuge for 30 min. Supernatants were collected and protein concentration was determined by using the Bradford kit (Pierce). Samples were subjected to SDS‐PAGE (4–12 % gels) and transferred onto Protran 0.2 NC nitrocellulose membranes (Amersham). Membranes were blocked for 1 h at room temperature with 5 % non‐fat milk or bovine serum albumin (BSA) in Tris‐buffered saline (TBST; 50 mm Tris**⋅**HCl and 150 mm NaCl, pH 7.5) containing 0.1 % Tween‐20 in phosphate‐buffered saline (PBS, pH 7.4), and probed with the indicated antibodies overnight at 4 °C. Detection was performed using horseradish peroxidase (HRP)‐conjugated secondary antibodies and enhanced chemiluminescence reagent.

**Ubiquitin enrichment**: For ubiquitylated protein capture, extract (400 μg) was used for pull down with HALO‐UBAUBQLN1 resin, as described previously.4c Halo‐tagged ubiquitin‐binding domains (UBDs) of UBQLN1 were incubated with HaloLink resin (200 μL, Promega) in binding buffer (50 mm Tris**⋅**HCl, pH 7.5, 150 mm NaCl, 0.05 % NP‐40) overnight at 4 °C. Halo Tube beads (20 μL) were added to neuronal or tissue lysates and incubated for 4 h at 4 °C. The beads were washed three times with lysis buffer containing 0.25 m NaCl and eluted by resuspension in 1×LDS sample buffer (20 μL) with 1 mm dithiothreitol (DTT).

**Flow cytometry analysis of mitochondrial membrane potential**: HeLa Cells were incubated with 20 μm niclosamide (Sigma–Aldrich) and AM85 (for synthesis, see the Supporting Information) for 40 min before trypsinization and collection. Oligomycin (Sigma–Aldrich) and antimycin (Sigma‐Aldrich) 20 μm were used as positive controls and incubated for 3 h before sample harvest. DMSO (Sigma Aldrich) was used at the same concentration as a control. After 10 min from the start of drug treatment, cells were treated with 100 nm Mito Tracker CMXRos (Cell Signaling Technology) for 30 min directly on wells. Drug wash out was performed by incubating trypsinized floating cells with 100 nm Mito Tracker CMXRos (Cell Signaling Technology) for 30 min at 37 °C in the absence of drug. All harvested cells were incubated for 5 min on ice after CMXRos incubation and then centrifuged and washed two times with a 1 % solution of BSA/PBS. Finally, cells were treated with a solution of 4′,6‐diamidino‐2‐phenylindole (DAPI; 1:200; 1 mg mL^−1^ DAPI, 50 μg mL^−1^ RNaseA in 1 % BSA/PBS) and transferred to FACS tubes for analysis. Samples were acquired by using a BD FACS Canto system and the results analysed by using FlowJo software.

**Statistical analysis**: Statistical analysis of groups with normal distributions was performed by means of one‐ or two‐way ANOVA followed by Holm–Sidak or Bonferroni post‐tests. Differences among groups were considered statistically significant if *p*<0.05. Data throughout the text are reported as average values±SEM, unless otherwise specified.

## Conflict of interest

*The authors declare no conflict of interest*.

## Supporting information

As a service to our authors and readers, this journal provides supporting information supplied by the authors. Such materials are peer reviewed and may be re‐organized for online delivery, but are not copy‐edited or typeset. Technical support issues arising from supporting information (other than missing files) should be addressed to the authors.

Supplementary
