# Supplementary material for: The Anthelmintic Drug Niclosamide and Its Analogues Activate the Parkinson's Disease Associated Protein Kinase PINK1
Source: Chembiochem. 2018 Jan 24;19(5):425–9. doi: 10.1002/cbic.201700500 (PMC5901409; doi:10.1002/cbic.201700500)
Supplement: Supplementary file 1 — Supplementary [file CBIC-19-425-s001.pdf]

CHEMBIOCHEM

Supporting Information

The Anthelmintic Drug Niclosamide and Its Analogues Activate the Parkinson's Disease Associated Protein Kinase PINK1

Erica Barini,[a] Ageo Miccoli,[b] Federico Tinarelli,[c] Katie Mulholland,[a] Hachemi Kadri,[b] Farhat Khanim,[d] Laste Stojanovski,[e] Kevin D. Read,[e] Kerry Burness,[f] Julian J. Blow,[c] Youcef Mehellou,*[b] and Miratul M. K. Muqit*[a, g]

cbic_201700500_sm_miscellaneous_information.pdf

The Anthelmintic Drug Niclosamide and its Analogues Activate the Parkinson's Disease Associated Protein Kinase PINK1

Erica Barini,[1] Ageo Miccoli,[2] Federico Tinarelli,[3] Katie Mulholland,[1] Hachemi Kadri,[2] Farhat Khanim,[4] Laste Stojanovski,[5] Kevin D. Read,[5] Kerry Burness,[6] Julian Blow,[3] Youcef Mehellou,[2,*] Miratul M. K. Muqit[1,7,*]

[1]MRC Protein Phosphorylation and Ubiquitylation Unit, School of Life Sciences, University of Dundee, Dow Street, Dundee DD1 5EH, UK.

[2]School of Pharmacy and Pharmaceutical Sciences, College of Biomedical and Life Sciences, Cardiff University, King Edward VII Avenue, Cardiff CF10 3NB, UK.

[3]Gene Regulation and expression department, School of Life Sciences, University of Dundee, Dow Street, Dundee DD1 5EH, UK.

[4]School of Biosciences, College of Life and Environmental Sciences, University of Birmingham, Edgbaston, Birmingham B15 2TT, UK.

[5]Drug Discovery Unit, School of Life Sciences, University of Dundee, Dow Street, Dundee DD1 5EH, UK.

[6]Division of Signal Transduction Therapy, School of Life Sciences, University of Dundee, Dow Street, Dundee DD1 5EH, UK.

[7]School of Medicine, University of Dundee, Dundee DD1 9SY, UK.

CONTENT

I. Antibodies and reagents (page 1)
II. Cell culture (page 2)
III. Synthesis of AM85-AM87 compounds (page 2)
IV. Supplementary figure S1 (page 3)
V. Supplementary figure S2 (page 4)
VI. Supplementary figure S3 (page 5)

I. Antibodies and reagents

The following primary antibodies were used: mouse monoclonal antibodies against Parkin (Santa Cruz), βI-tubulin (Sigma), β-actin (Sigma), GAPDH (Santa Cruz), PSD95 (Cell Signaling), synaptophysin (cell signalling), CISD1 (Proteintech). Horseradish-peroxidase (HRP)-conjugated secondary antibodies (Sigma) were used. Anti-Parkin phospho-Ser65 rabbit monoclonal antibody was raised by Epitomics in collaboration with the Michael J Fox Foundation for Research.

Stock solutions of niclosamide (Sigma), Antimycin A (Sigma) Oligomycin A (Sigma) were used for experiments in vitro. Unless otherwise specified, general reagents and chemicals were from Sigma and cell culture reagents were from Invitrogen.

II. Cell culture

HeLa cells stably expressing untagged Parkin WT and PINK 1 KO, were cultured using DMEM (Dulbecco's modified Eagle's medium) supplemented with 10% FBS, 2 mM L-glutamine, 1×penicillin/streptomycin. To uncouple mitochondria, cells were treated with 10 µM Antimycin (Sigma) and 1 µM Oligomycin dissolved in DMSO for 3 h. To express Parkin, cell transfections were performed using polyethylenimine (Polysciences) according to the manufacturer's instruction.

Primary cortical neurons were isolated from the brain of WT embryos of either sex at E16.5. Embryonic cortices were collected in HBSS, and cells were dissociated by incubation with trypsin (GIBCO) at 37°C. Cells were then diluted in Neurobasal medium containing B27 supplement, Glutamax, penicillin/streptomycin and plated at a density of 6.0 × 106 cells/well on 6-well plates coated with 0.1 mg/ml poly-L-lysine (PLL; Sigma). Neurons were cultured at 37 °C in a humidified incubator with 5% CO_2. Every 7 days, the medium was replaced with fresh medium containing B27. Neurons were treated with 30 µM niclosamide and analogues for 1h at 37 °C.

III. Synthesis of AM85-AM87 compounds

To a stirring solution of the relevant salicylic acid (1 eq.) in dry THF, PyBop (1.2 eq.) and triethylamine (3 eq.) were added. After 10 min, the relevant aniline (1.5 eq.) was added to the cloudy white suspension and stirred at room temperature for 12 h. The reaction mixture was then concentrated under reduced pressure to afford a yellow oil, which was subsequently dissolved in water and extracted in ethyl acetate. The combined organic layers were dried ($MgSO_4$), filtered and concentrated under reduced pressure to form a viscous orange oil. Flash column chromatography employing ethyl acetate:hexane (1:1) was used to yield the desired AM compounds as off-white solids.

5-Bromo-N-(4-bromophenyl)-2-hydroxybenzamide (AM85). Yield 10%. ^{1}H NMR (500 MHz, DMSO): δ 11.73 (s, 1H, OH), 10.49 (s, 1H, NH), 8.03 (d, J = 2.5 Hz, 1H), 7.69 (d, J = 8.9 Hz, 2H), 7.57 (m, 3H), 6.97 (d, J = 8.8 Hz, 1H). ^{13}C NMR (126 MHz, DMSO): δ 165.36, 157.53, 137.98, 136.31, 132.39, 131.77, 123.10, 120.92, 119.97, 116.46, 110.59. HRMS m/z $[M+H]^+$ calcd. for $C_{13}H_{10}NO_2Br_2$: 369.9078, found: 369.9067. Anal. Calcd for $C_{13}H_9NO_2Br_2$: C, 42.08; H, 2.44; N, 3.77. Found: C, 41.93; H, 2.61; N, 3.93.

3,5-Dibromo-N-(4-bromophenyl)-2-hydroxybenzamide (AM86). Yield 9%. ^{1}H NMR (500 MHz, DMSO): δ 12.75 (s, 1H, OH), 10.71 (s, 1H, NH), 8.26 (s, 1H), 8.01 (s, 1H), 7.67 (d, J = 8.6 Hz, 2H), 7.59 (d, J = 8.5 Hz, 2H). ^{13}C NMR (126 MHz, DMSO): δ 167.09, 156.71, 139.09, 137.27, 132.07, 130.26, 124.06, 119.10, 117.41, 112.93, 110.39. HRMS m/z $[M+H]^+$ calcd. for $C_{13}H_9NO_2Br_3$: 447.8183, found: 447.8184. HPLC (8 min), t_R = 5.21 min, purity: 97%.

3,5-Dibromo-2-hydroxy-N-phenylbenzamide (AM87). Yield 15%. ^{1}H NMR (500 MHz, DMSO): δ 13.01 (s, 1H, OH), 10.65 (s, 1H, NH), 8.31 (d, J = 2.2 Hz, 1H), 8.02 (d, J = 2.2 Hz, 1H), 7.68 (d, J = 7.7 Hz, 2H), 7.41 (t, J = 7.9 Hz, 2H), 7.21 (t, J = 7.4 Hz, 1H). ^{13}C NMR (126 MHz, DMSO): δ 167.18, 156.98, 138.91, 137.87, 130.26, 129.21, 125.40, 122.23, 118.88, 113.14, 109.71. HRMS m/z $[M+H]^+$ calcd. for $C_{13}H_{10}NO_2Br_2$: 369.9078, found: 369.9087. Anal. Calcd for $C_{13}H_9NO_2Br_2$: C, 42.08; H, 2.44; N, 3.77. Found: C, 41.91; H, 2.37; N, 3.71.

IV. Supplementary figure 1

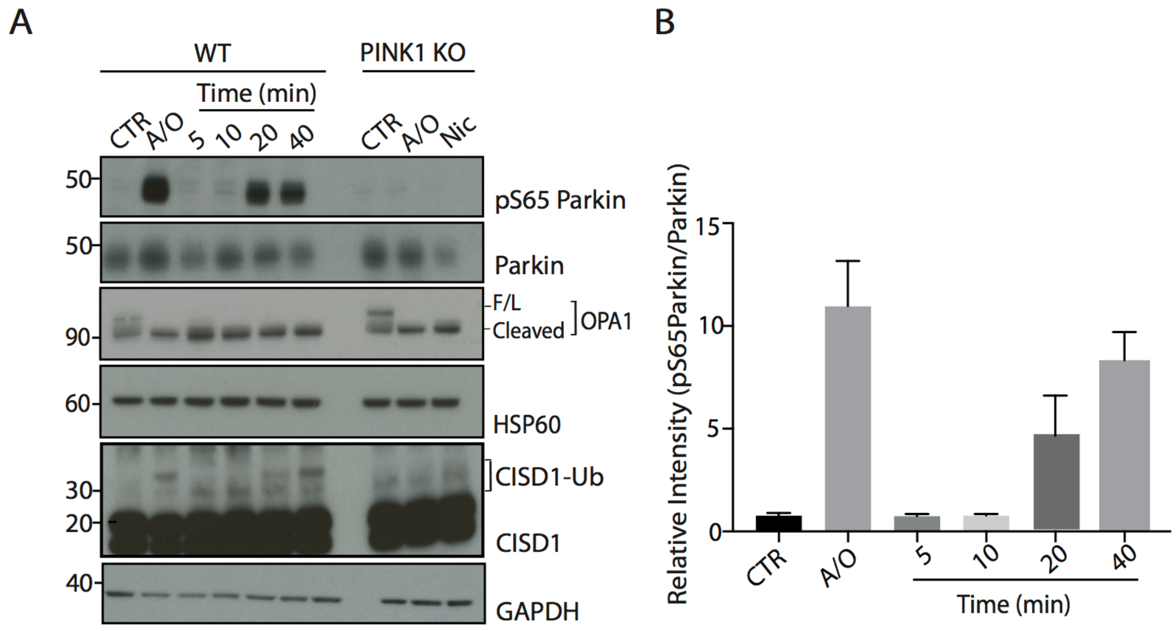

Supplementary 1. Niclosamide activates PINK1 after 20 minutes treatment. A) Niclosamide-induced Parkin activation is PINK1 dependent and can be detected after 20 min of *in vitro* stimulation. HeLa cells transfected with Parkin were stimulated with either a combination of antimycin A and oligomycin A (A/O) for 3 h or with 10 µM of niclosamide (Niclo) for 5, 10, 20, 40 min. Detection of Parkin S65 phosphorylation (pS65Parkin), Parkin, Full length (F/L) and cleaved OPA1, CISD1 ubiquitylation (CISD1-Ub) can be detected in niclosamide stimulated HeLa cells at different time points. GAPDH and HSP60 were used as loading controls. **B**) Quantitative analysis of Parkin S65 phosphorylation after niclosamide treatment at different time points (5, 10, 20, 40 min). Bars represent the average ratio ± SEM of two independent experiments and the value is a ratio between pS65 Parkin and total Parkin.

V. Supplementary figure 2

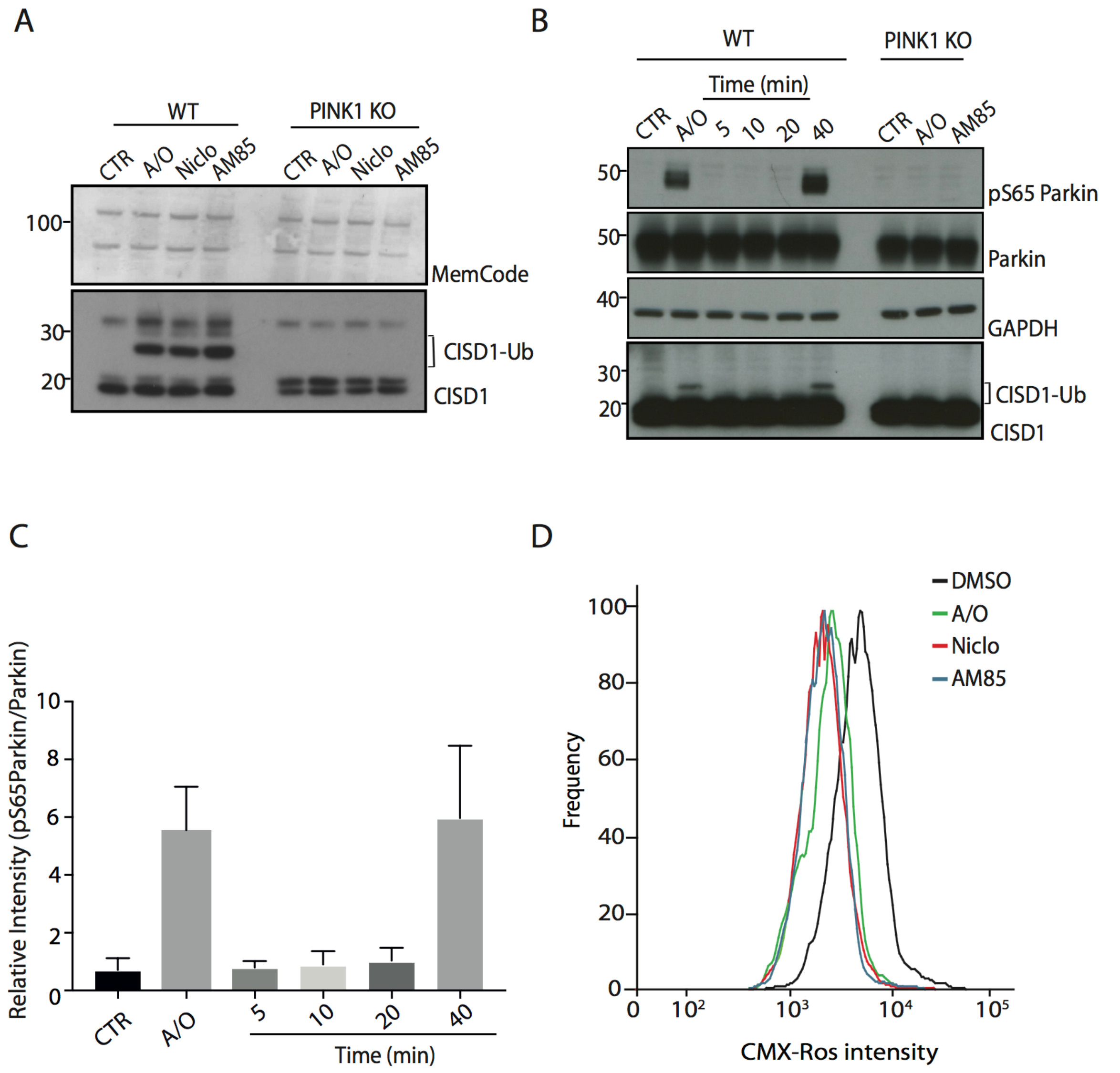

Supplementary 2. AM85 activates PINK1 and uncouples mitochondria after 40 minutes of treatment. A) CISD1 ubiquitylation after niclosamide and AM85 treatment is PINK1 dependent. Wildtype (WT) and PINK1 knockout (Pink1 KO) HeLa cells transfected with Parkin were stimulated with a combination of 10 µM antimycin A and 1 µM oligomycin A (A/O) for 3 h, 10 µM niclosamide (Niclo) and 10 µM AM85 for 40 min. CISD1 ubiquitylation (CISD1-Ub) was detected by western blotting. **B**) Time course of AM85 in HeLa cells. Detection of Parkin S65 phosphorylation (pS65 Parkin), Parkin, Full length and cleaved OPA1 and CISD1 ubiquitylation (CISD1-Ub) upon different time points of AM85 treatment (5, 10, 20, 40 min). **C**) Quantitative analysis of Serine 65 phosphorylation in response to AM85 treatment at different time points. Bars represent the average ratio ± SEM of 2 independent experiments and the value is a ratio between pSer65 Parkin and total Parkin. **D**) Niclosamide and AM85 uncouple mitochondria in HeLa cells. Exemplary FACS graph of CMXRos fluorescence intensity in Hela cells treated on site. Hela cells were treated on site with antimycin A and oligomycin A (A/O, green), niclosamide (Niclo, red), AM85 (blue) and the vehicle, DMSO (black).

VI. Supplementary figure 3

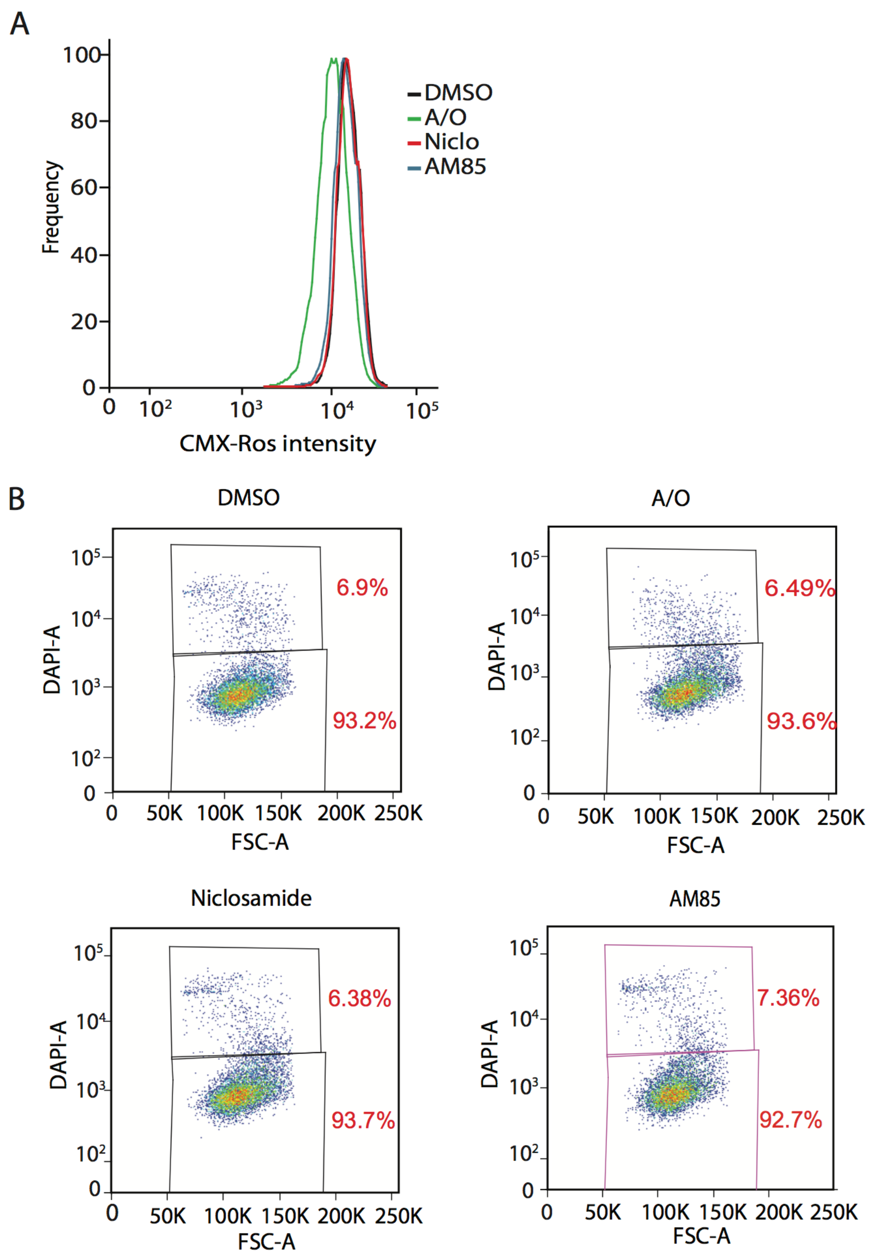

Supplementary 3. Mitochondrial membrane potential is restored upon wash out of AM85 and niclosamide. A) Exemplary FACS curve of CMXRos fluorescence intensity in Hela cells subjected to 30 min washout treatment. Hela cells treated with Antimycin A/ Oligomycin A (A/O, green curve), niclosamide (Niclo, red curve), AM85 (blue curve), normalized to the vehicle, DMSO (black curve). **B**) Niclosamide and AM85 do not induce cell death. Percentage of alive (bottom percentage) and dead cells (upper percentage) after niclosamide (bottom left square) and AM85 (bottom right square) treatment compared to DMSO (upper left square) and A/O (upper right square) treated cells. FSC-A (forward scatter area).
